# A Novel, Low-Cost, Non-laboratory Training Model for Neurosurgical Microdrilling Skills: A Proof-of-Concept Study

**DOI:** 10.7759/cureus.99183

**Published:** 2025-12-14

**Authors:** Ntenis Nerntengian, Theodosis Birbilis, George Tokas, Alexey Ivanchenko, Andrea Hajduk, Oliver W Sakowitz

**Affiliations:** 1 Neurological Surgery, RKH Klinikum Ludwigsburg, Ludwigsburg, DEU; 2 Neurological Surgery, Democritus University of Thrace, Alexandroupolis, GRC; 3 Neurological Surgery, SLK-Kliniken Heilbronn, Heilbronn, DEU

**Keywords:** eggshell peeling, low-cost model, microdrilling, nail drill, psychomotor skill, resident training, surgical simulation, training at home, walnut drilling

## Abstract

Introduction

Surgical simulation is essential in neurosurgical resident training for dexterity development in a risk-free environment. Microdrilling is a fundamental neurosurgical skill. Practicing this skill usually requires specially equipped facilities with a biological or synthetic material and thus has limited availability. Up-to-date computerized virtualization cannot fully replace the training of hand-eye sensorimotor coordination. The aim of this study is to develop and preliminarily evaluate a low-cost, lab-free proof-of-concept microdrilling model using walnuts, a nail drill, and surgical loupes and to explore performance differences between neurosurgical residents and consultants.

Materials and methods

We used a surgical loupe with LED light and 3.5x magnification, walnuts, and a battery-powered nail drill. Some walnuts were drilled from the flat surface toward the convoluted surface while preserving the outer skin, whereas in others, the skin was drilled away from the kernel to maintain the natural contour of the walnut. Drilling skills were compared between residents and consultants.

Results

When drilling from the flat surface to the convoluted surface, the truncated cone-shaped drill bit was initially used to flatten the irregular parts of the walnut. The acorn-shaped drill bit and the conical drill were then used to further reach the skin and leaving it intact as in the eggshell peeling technique. For drilling of the skin along the kernel’s surface to maintain its contour, the conical bit was used for precision. While drilling the kernel towards the convoluted surface, no perforations were made after the first attempt by consultants and after two attempts by the residents. All participants reported a subtle loss of resistance just before reaching the skin.

Conclusion

Despite its limitations, our model offers an easily reproducible concept that allows the basic principles of microdrilling to be practiced on a delicate substrate without risk of infection and without requiring a surgical high-speed drill or laboratory access, and it could potentially benefit the drilling skills of residents.

## Introduction

The use of high-speed drills for bone removal and associated peeling techniques under magnification (“egg-shelling”) is an essential neurosurgical skill, required especially in skull base procedures [1-3]. Optimizing this technique improves the quality, safety, and ease of both cranial and spine procedures. Due to time constraints in modern medicine, achievement of the required psychomotor skills has become increasingly difficult. It has been suggested that utilizing various simulating models parallel to the classical apprenticeship and clinical practice is of utmost importance [4-7]. Education using simulation allows practicing of motor skills in a safe and controlled environment [7-10]. The majority of the existing literature on practicing bone drilling skills includes various models of the temporal bone, either made out of synthetic materials or virtual models [2,3,6,7,9-12], most likely because of its anatomical complexity and its infrequent involvement in various skull base approaches. Cadaveric material still remains the optimal choice for the enhancement of bone drilling skills outside of the operating room [5,11,13]. The limited availability of cadaveric material [2,3,6] and the high cost of the simulation hardware and software combined with the lack of proper facilities obviates supplemental training for the majority of neurosurgery residents. The aim of this study is to develop and preliminarily evaluate a proof-of-concept model that is low-cost, lab-free, and easily accessible to practice bone drilling and the eggshell peeling technique under magnification utilizing surgical loupes, walnuts, and a battery-powered nail drill as well as to explore performance differences between neurosurgical residents and consultants. The thin walnut skin provides a delicate and easily damaged surface, making it a suitable substrate for practicing the precision required in microdrilling. Although microsuturing can be practiced in a relatively affordable manner and unrestricted to a lab environment (e.g., with a pair of surgical loupes, jewelers’ forceps, microsutures, plastic gloves and/or microtubes from synthetic material), to our knowledge, a low-cost model for practicing drilling techniques aside the need for a laboratory and a surgical high-speed drill has not yet been reported. 

## Materials and methods

**Figure 1:**
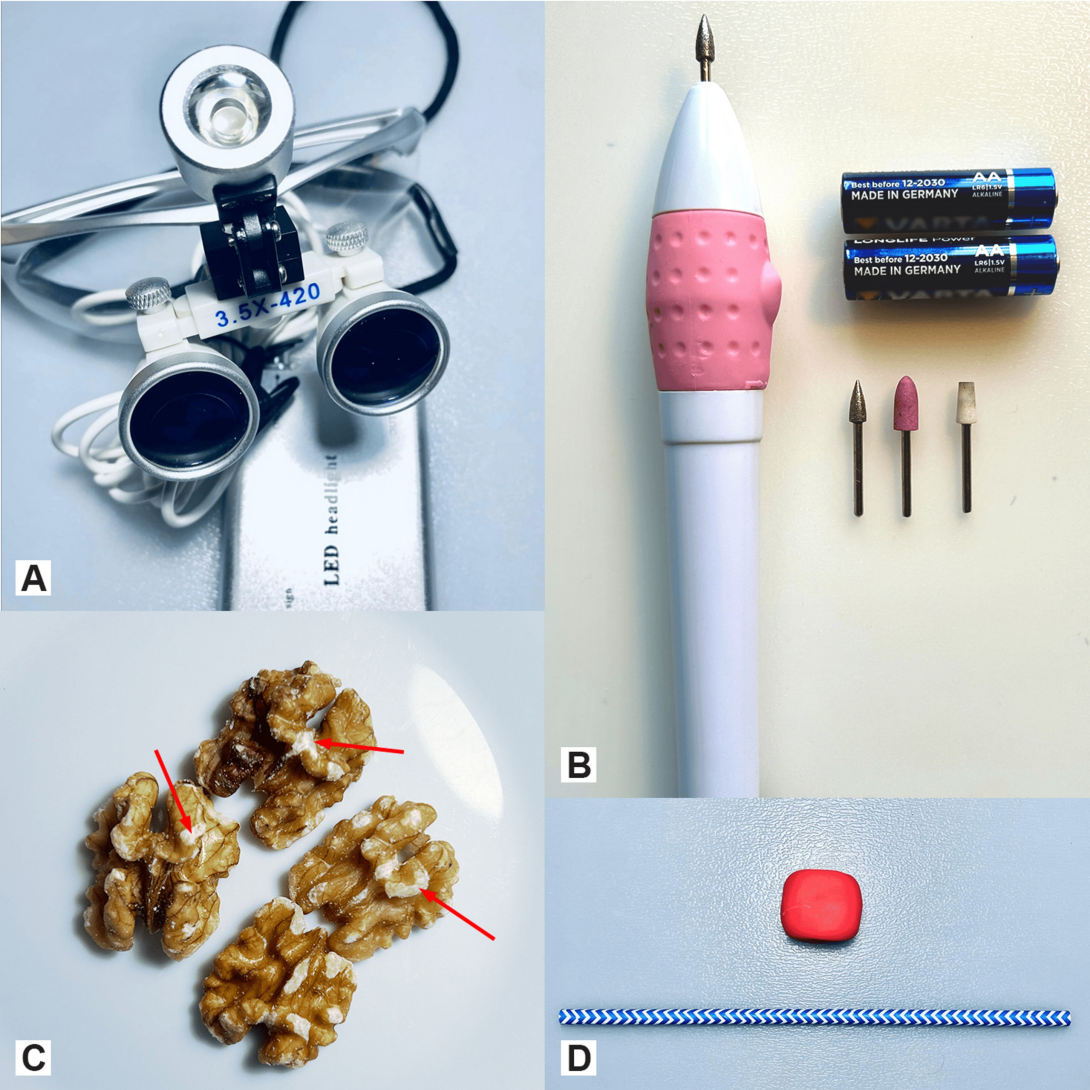
Materials used A. Surgical loupes. B. The battery-powered nail drill with three different types of drill bits: from left to right, the metallic sharp conic shaped, the silicon acorn-shaped and the felted wool truncated cone-shaped. C. Walnuts, the red arrows show the regions where naturally the thin walnut skin is absent. D. The modelling dough and the drinking straw.

To set up a training model, we used surgical loupes with a 3.5x magnification at a working distance of 420 mm and an attachable external LED-light source, walnut kernels with their skin, a nail drill powered by two type AA 1.5 V batteries with three different types of drill bits, a 1 mm metallic sharp conic-shaped drill bit, a 3 mm felted wool truncated cone-shaped drill bit and a 5 mm silicon acorn-shaped drill bit, a drinking straw and modelling dough (Figures 1A-1D).

**Figure 2:**
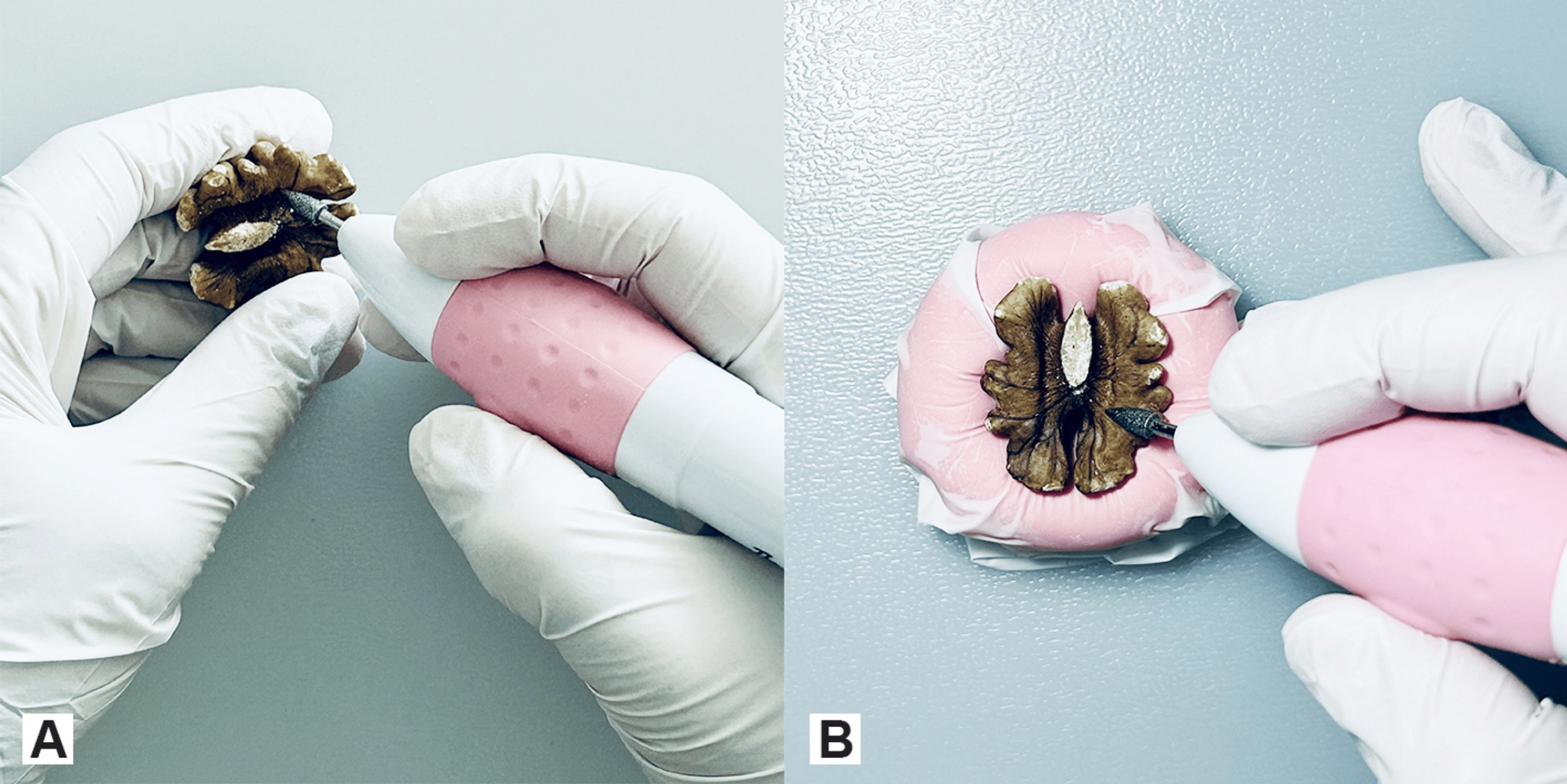
Positioning of the walnut A. Holding the walnut in the hand trains the gentle tactile handling while drilling from the inside to avoid breakage of the thinned parts. B. Placing the walnut on a modelling dough provides stability.

The surgical loupes and the battery-powered nail drill with its drill bits can be easily obtained from various online shops. The batteries, the drinking straw, and walnut kernels are available both online and in various local markets. The total cost of all the above-mentioned equipment was approximately fifty euros. The walnut can either be held freely on the non-dominant hand or placed and pressed in a modelling dough, which is covered with a single layer of plastic gloves (Figures 2A, 2B) to protect the modelling dough from the chips or melted walnut kernel produced while drilling, in order to be reused.

**Figure 3:**
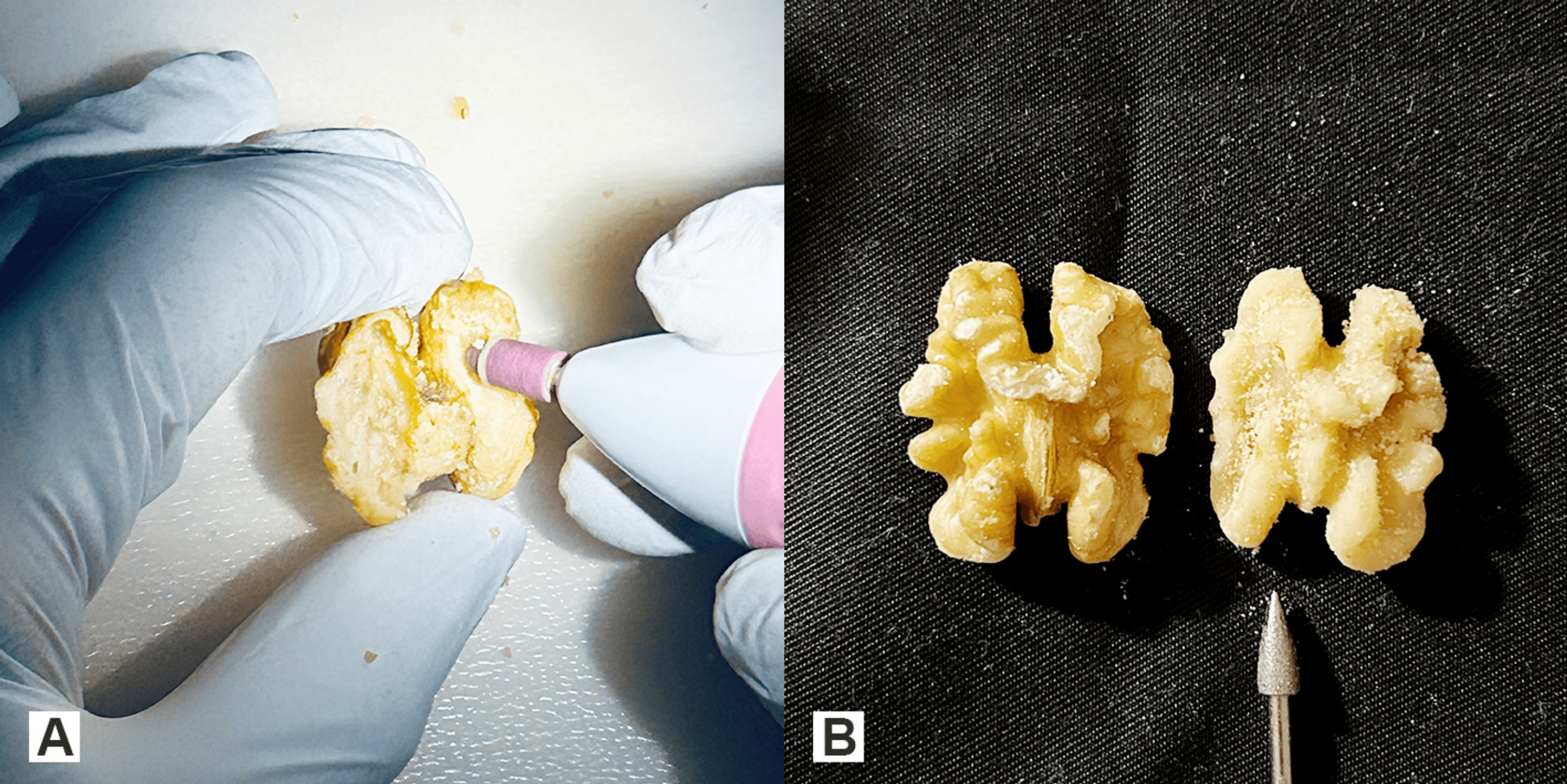
The two ways of drilling practice A. Drilling the walnut kernel. The image shows a walnut drilled on both sides beginning from the flat towards its convoluted surface with the acorn-shaped drill bit without perforating the kernel skin. B. The skin of the convoluted surface on the right side is drilled away with the sharp, pointed bit, preserving the natural convolutions. Not drilled walnut on the left side for comparison.

The trainee can either choose to drill the walnut kernel beginning from the flat towards the convoluted surface without causing a perforation to the delicate walnut kernel skin or to drill away the walnut kernel skin from the walnut kernel without violating the contour of the kernel (Figures 3A, 3B). 

The produced debris while drilling is regularly and gently blown away with the drinking straw. This study was primarily intended to demonstrate the feasibility and practical setup of a low-cost, lab-free microdrilling model rather than to evaluate specific performance parameters. Three residents in various years of residency (R1-junior resident, R2-resident in the intermediate phase of the residency and R3-senior resident) and three consultant neurosurgeons with similar surgical experience S1-3 were given each time a relatively similar shaped piece of walnut for a total of five attempts to completely drill the kernel of that piece from beginning from the flat towards its convoluted surface without penetrating the skin of the kernel. Residents had typical prior exposure for their training level and consultants had several years of independent drilling experience. For each attempt, we recorded the number of perforation points, and we compared the mean values per person per attempt between residents and consultants, as well as the total number of perforations between the R1 and the combined R2 and R3 group. All perforations were recorded by the same independent observer, a consultant, using the surgical loupes. Given the small sample size, we report these results descriptively without formal statistical testing. 

## Results

We divided the drilling practice into two different scenarios, each with its respective feature.

Drilling the kernel from the flat towards the convoluted surface

**Figure 4:**
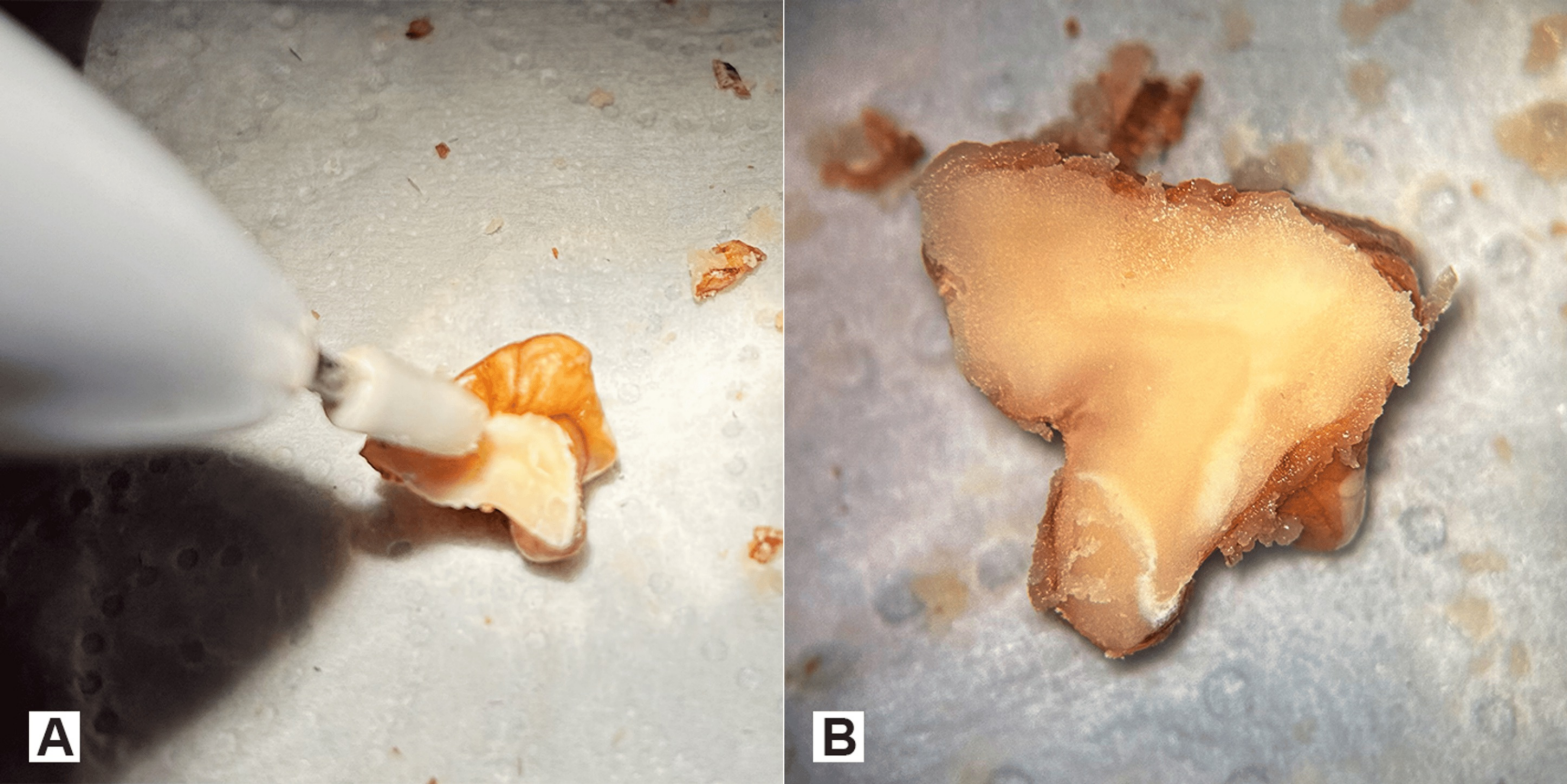
The use of truncated cone-shaped drill bit A. Uneven parts of a walnut part can be flattened out with this drill bit. B. Smooth surface of the walnut kernel enlarges the available surface to drill further with the acorn-shaped drill bit.

While drilling the kernel from the flat towards the convoluted surface of the walnut, the truncated cone-shaped drill bit can initially be used to flatten the irregular parts of the walnut piece and take out big chunks (Figures 4A, 4B) to lay the ground for further finer drilling into the deeper wrinkles of the walnut with the acorn shaped and the sharp conic shaped drill bit.

**Figure 5:**
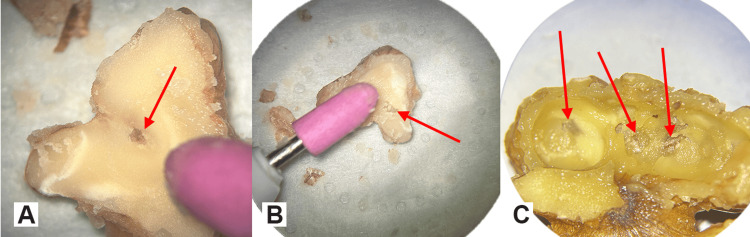
The use of the acorn-shaped drill bit Reaching the walnut skin making smooth circular movements. Red arrows in A, B and C indicate the regions where the skin has been reached

The acorn-shaped bit can then be utilized to gradually expose the walnut skin making small circular movements towards the convoluted surface and removing the round-shaped kernel pieces as in the eggshell-peeling technique, which is used in various operations, for example while doing burr holes, skeletonizing a sinus or performing laminectomies (Figures 5A-5C).

**Figure 6:**
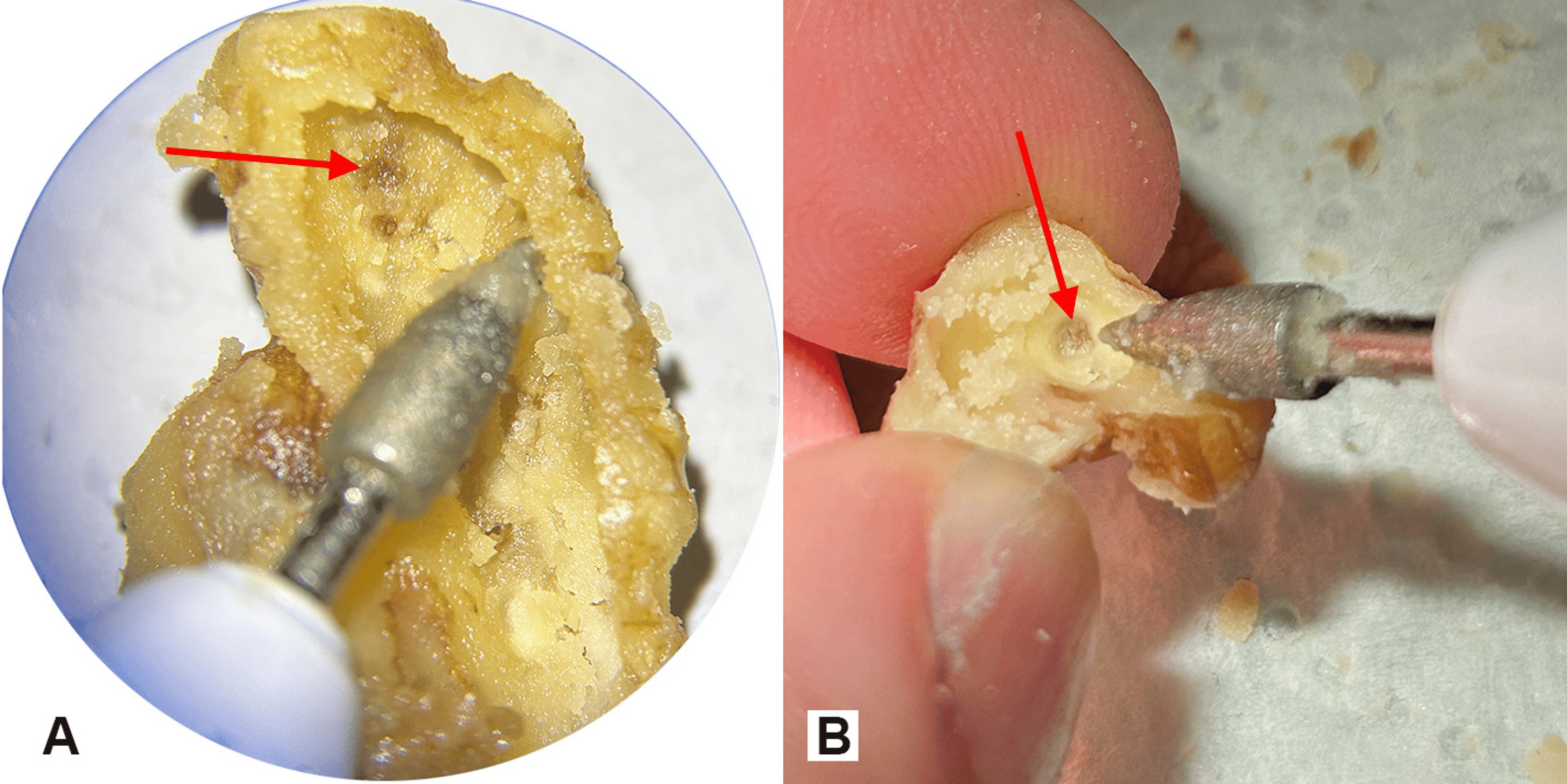
The use of the sharp-tipped conical bit With its thin tip, this bit can reach the walnut skin of deeper convolutions with fine movements (red arrows).

The conical drill allows refined drilling exposing the kernel skin which lies deeper in the convolutions of the walnut kernel owing to its fine and long tip after having removed bigger kernel chunks with the acorn-shaped drill bit (Figures 6A, 6B).

**Figure 7:**
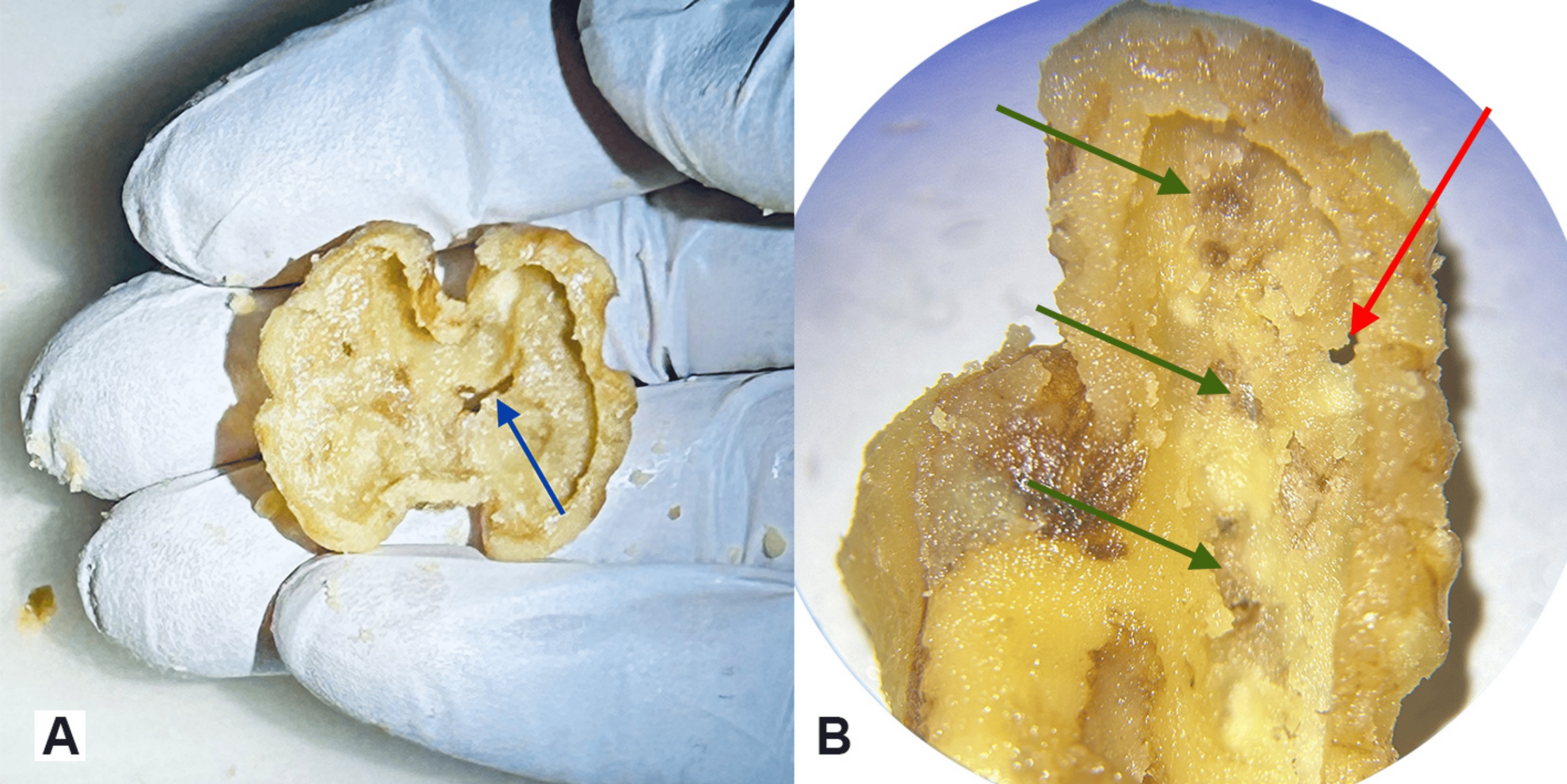
Specimens with skin breaches A. A walnut drilled-carved out on its both “hemispheres” leaving just the kernel skin. The blue arrow demonstrates a “pseudobreach” in a region where naturally no walnut skin was present. B. The breach at this specimen is true and was caused by the participant during drilling (red arrow). Green arrows show the regions where the skin is intact and properly exposed.

As seen in Figure 1C, walnut kernel skin is missing at a few parts of the convoluted surface of the walnut; this can create some “pseudobreaches” of the skin when drilling from the inside out (Figures 7A, 7B).

**Figure 8:**
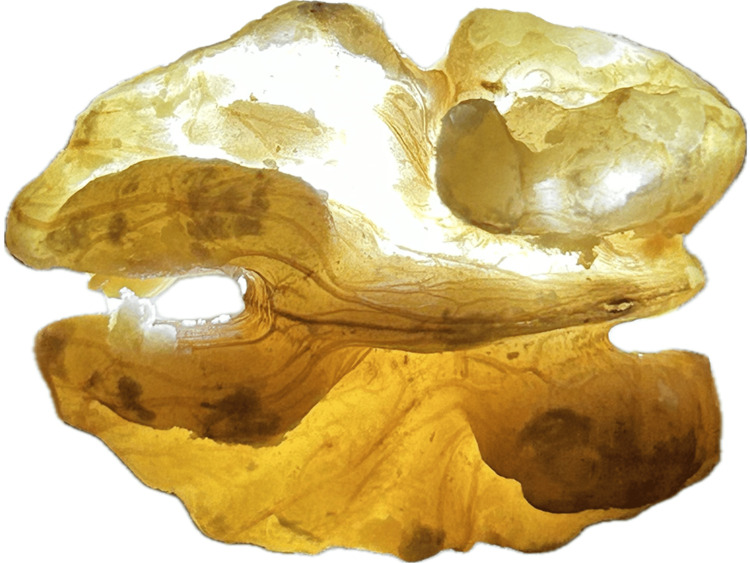
The tenuous walnut skin exposed to the light source After complete removal of the walnut kernel, the remaining skin has a thickness of rice paper and is almost transparent to light, revealing its vessel pattern.

After successful drilling of the kernel, the remaining skin is so thin that when exposed to the light source it should appear almost transparent like a rice paper and the vessel pattern (darker brown lines on the skin) of the kernel skin can be recognized (Figure 8).

**Figure 9:**
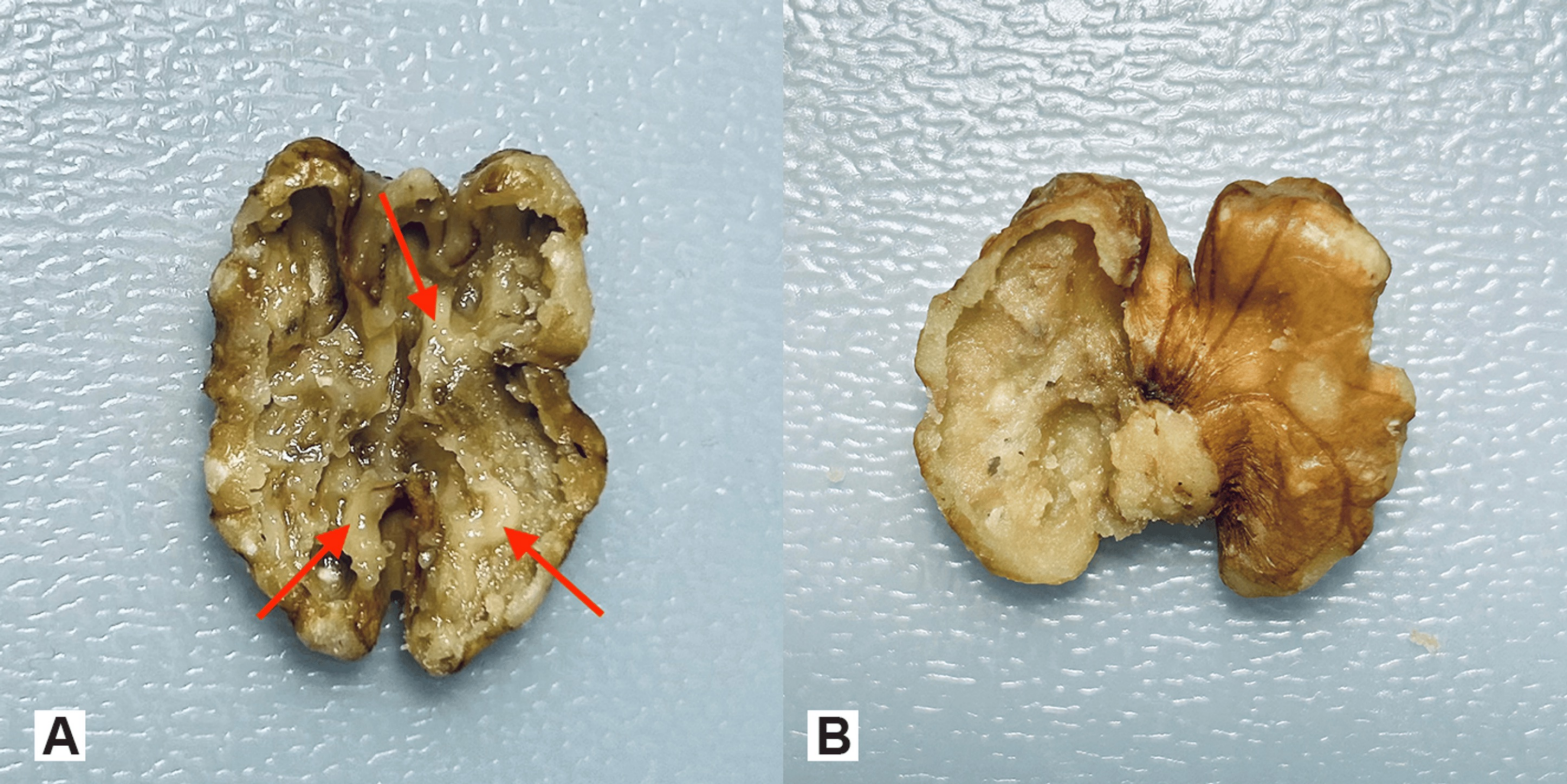
Removal of debris Regularly blowing off the debris is important to keep a clear view. A. For demonstration purposes debris in this specimen was intentionally not removed as they developed and thus it became sticky and glued together (red arrows show some of the areas with sticky debris). B. Specimen drilled while debris is frequently removed generates a clean field.

The walnut kernel is rich in fat and proteins [14]. These are heat-sensitive substances, which, in the process of drilling the walnut kernel, generate a debris that can get sticky due to heat by friction if not removed frequently and obstruct the visual field. For this reason, one has to perform intermittent drilling to prevent high heat and blow away the debris regularly with a drinking straw as they build up not allowing them to glue together (Figures 9A, 9B).

Drilling away the skin from the kernel 

**Figure 10:**
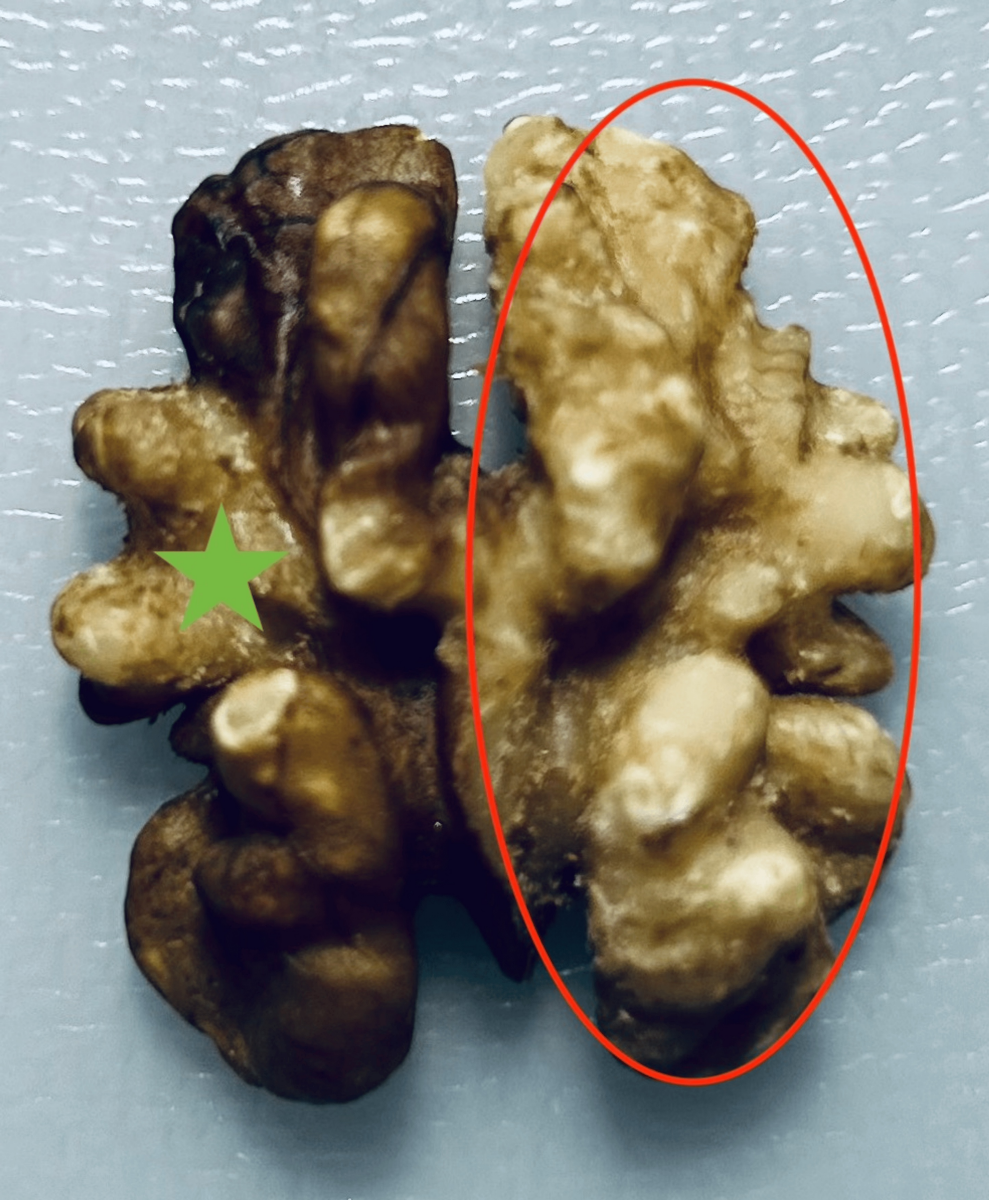
Drilling of the skin on the convoluted surface The skin of a wedge-shaped area on the left (green asterisk) and the skin of the complete right side of the walnut are drilled away (red circle). Note that the convoluted contour is preserved.

The sharp conic bit is used when removing the kernel skin from the kernel while trying to preserve the texture of the walnut kernel either on the convoluted or its flat side. To achieve this, a small circular area of the kernel skin is initially gently drilled away from the kernel without pushing the drill bit into the kernel performing gentle circular motions. From this small area of exposure, the trainee can expand the peeling area radially towards the desired direction, delicately peeling the skin away from the kernel using only the tip of the sharp conic drill bit by steadily fine-tuning the amount of pressure exerted in order to preserve the contour of the walnut kernel especially on its convoluted surface (Figure 10).

**Figure 11:**
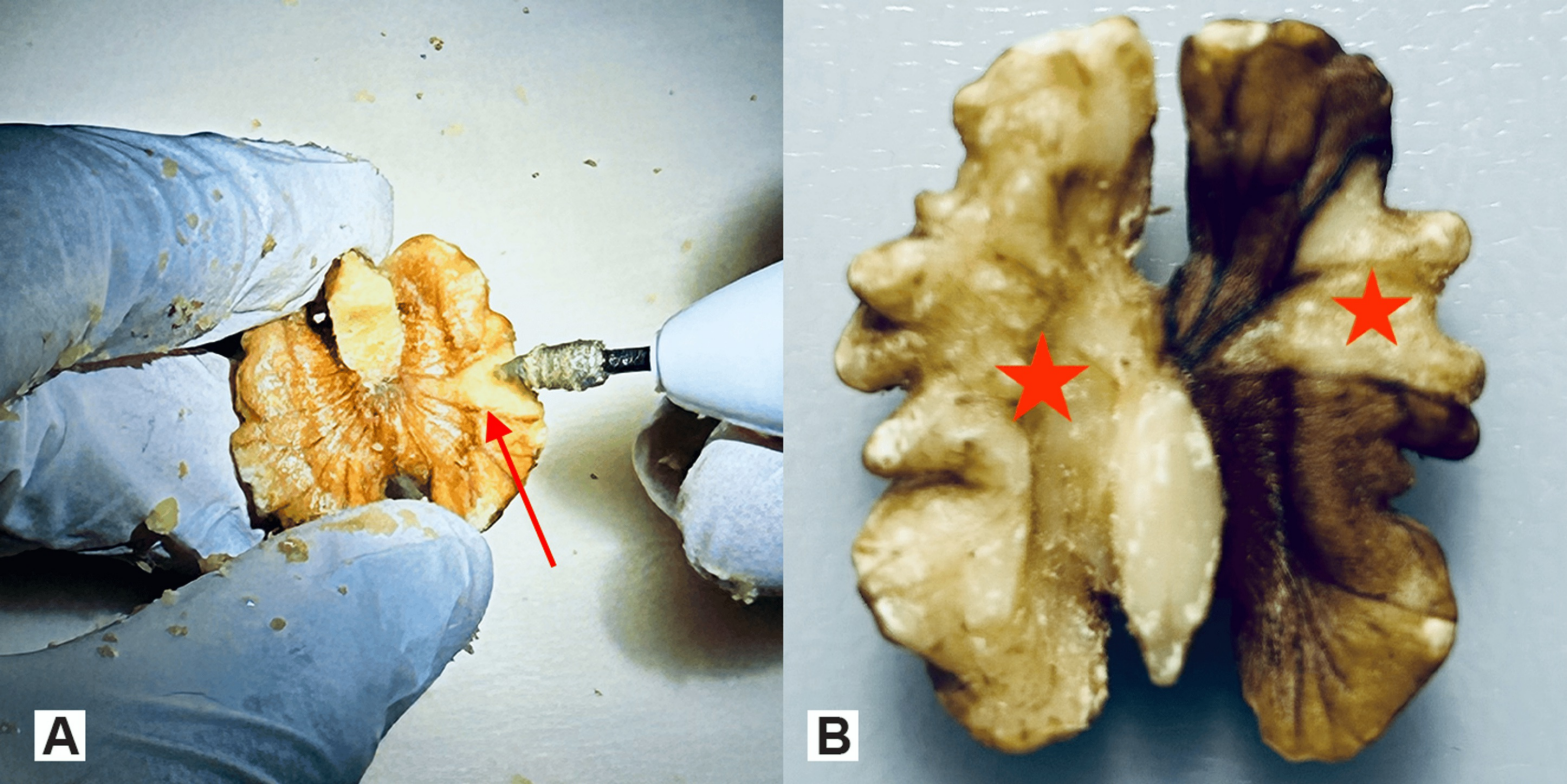
Drilling of the skin on the flat surface A. A wedge-shaped area of walnut skin between the two dark lines is removed (red arrow) with the sharp metallic drill bit without further penetrating into the kernel. B. Regions with the red asterisks represent the areas where the thin walnut skin is drilled away. Note that the contour of the kernel is preserved.

As an additional practice step, a triangular area on the flat surface of the walnut can be peeled between two dark lines of the kernel skin (Figures 11A, 11B).

Comparison of the results between residents and consultants while drilling a walnut piece from the flat towards the convoluted surface

All of the participants adopted quickly and well to the exercise with none of the residents creating any perforation after the second attempt and none of the consultant neurosurgeons after the first attempt (Table 1).

**Table 1:** Number of perforations in attempts Nr. 1 to 5 made by residents and consultants

		Number of perforations					
Participants		R1	R2	R3	S1	S2	S3
Attempt No.	1	3	1	1	0	1	1
	2	2	1	1	0	0	0
	3	0	0	0	0	0	0
	4	0	0	0	0	0	0
	5	0	0	0	0	0	0

Residents showed more perforations in the first attempts, while consultants reached zero perforations earlier. In attempt 1, residents had a mean of 1.67 perforations (range 1 to 3) compared with 0.67 (range 0 to 1) in consultants. In attempt 2, residents had a mean of 1.33 (range 1 to 2), whereas consultants had no perforations. By attempt 3, all participants achieved zero perforations and maintained this in subsequent attempts. An exploratory comparison of cumulative perforations suggested that R1 had more breaches than the combined R2 and R3 group. These observations are descriptive as no inferential testing was performed due to the very small sample size. All of the participants reported a subtle loss of resistance after drilling the last bit of the kernel just before the exposure of the harder walnut skin while drilling the kernel from the inside out; roughly mimicking the loss of resistance when the epidural space is reached. This phenomenon may be attributed to the pectin layer at the interface between the kernel and the skin, which binds tightly to the skin but loosely to the kernel [14]. 

## Discussion

Importance of bone drilling under magnification and challenges in practicing 

Bone drilling under magnification and eggshell peeling technique is a key neurosurgical skill broadly required not only in complex skull base surgery [1,3,11] but also in simple spine procedures. Sufficient case exposure and practice lay the foundation for the acquisition of any surgical skill. There is consensus that honing microdrilling dexterities beyond the confines of the operating theater with computer simulations, animal models, cadavers and 3D printed models in a controlled and patient-safe manner, is of a paramount importance [1,2,5-10]. Medicolegal disquietudes, working hour restrictions, and lack of structured residency training [4,5] in contemporary medical practices, low volume of complex skull base cases which residents encounter [12], reinforce the relevance of supplemental exercising of the fine bone drilling as it requires a high degree of precision and accuracy to avoid damage to vital structures. 

Existing training models

Training in a laboratory with cadaveric material is, in terms of realism and accuracy, the ideal method for practicing surgical skills [5,13,15]. However, the limited availability, lack of facilities, and economic issues restrict the opportunity for cadaveric training [2,3,6,11]. Furthermore, it bears the risk of getting in contact with known and unknown infectious agents [16]. Animal models can also be utilized for bone drilling in a laboratory setting. The use of a pig skull for temporal bone drilling has been proposed because of its lower cost than human cadavers and sufficient resemblance to human anatomy [13]. Well-equipped medical institutions can even provide an in vivo porcine training model, which would allow the trainee, among various other microneurosurgical maneuvers, to perform bone drilling with the surrounding structures pulsating and bleeding [5]. Drilling the bone in chicken wings under magnification with a high-speed drill, after preparing and preserving the soft tissue around it, is an economically feasible method to practice microdrilling [1]. Cokluk has also described the use of walnut in a microdrilling model, but in his model, a surgical high-speed drill is utilized to drill the hard walnut shell with protection of the inner membranous structure beneath the shell under a surgical microscope in a laboratory setting [17]. Okuda et al. proposed a low-cost concept with a skull model and a chicken egg placed at the sella turcica for training endoscopic endonasal transsphenoidal surgery in which fine drilling skills are enhanced by drilling the eggshell with a high-speed drill while keeping the fragile shell membrane intact [15,18]. Computer-based simulations and virtual reality are significant aids for complementary enhancement of surgical dexterities, along with assisting better comprehension of complex anatomical relationships, yet they are expensive and lack tactile feedback [8,11]. Artificial 3D bone models made from synthetic materials can be expensive [9] and although close enough, may still lack the ability to duplicate the natural tactile feedback of a bone in its full extent [3,12]. Integration of soft tissue made out of different synthetic materials and of virtual soft tissue into the 3D printed bone models, coupled with technically sophisticated haptic devices, which can also manipulate structures in pure virtual reality models [3,9,10], offers an additional modern method to improve bone drilling dexterities. 

Benefits and practical value of a simple home-based training model

Surgical simulation and psychomotor training have become an essential part of neurosurgical resident training, as they enable practice and dexterity development in a risk-free environment. Not all institutions are equipped with a cadaver or a wet lab in which training can be done on biological materials. Additionally, not all neurosurgical clinics can provide one or more additional high-speed diamond drills for practice purposes. Realistic simulators are high-cost, and attending cadaveric courses alone does not provide the necessary continuity to regularly practice challenging maneuvers before applying them in the operating theatre. Mastering the eggshell peeling technique requires both cognitive and kinesthetic learning. The walnut kernel with its skin is a delicate substrate on which competency in bone microdrilling principles could be enhanced. Though lacking the realistic hardness of the bone and the high speed of the surgical drill, our concept provides a low-cost, easily reproducible, inanimate model that can nurture the skills of the trainees without the danger of infection and from the comfort of their homes. Conveniently obtainable simulation models could provide junior residents with experience as well as confidence before implementing the actual procedures on patients in order to achieve safer techniques and better surgical outcomes, especially when the resources are limited [19,20]. Our model can also function as a valuable antecedent step to improve drilling skills prior to cadaveric courses, where the real bone tissue has to be handled with utmost care. This could potentially enhance the performance of the trainee when attending these expensive courses by providing the opportunity to optimize the training benefit.

Limitations 

This study has several limitations. The sample size was very small, which is why the results are presented descriptively and without statistical testing. The nail drill is a basic battery-powered device that cannot reach the speed of a surgical drill, and the walnut does not match the hardness of real bone. The authors also recognize that different walnut types purchased by trainees may vary in skin thickness and in the consistency of the kernel, but this would mainly require a short adaptation to the drilling characteristics and does not pose a major limitation for the reproducibility of the model. Larger studies are needed to test the model with more participants and to provide external validation and long-term assessment. Simple forms of skill assessment could be added in future work, such as trainees recording their own time to completion and rating how helpful the model is for real operating room practice on a five-point scale from “not helpful at all” to “slightly helpful,” “moderately helpful,” “fairly helpful” and “very helpful” after training with the model for a given period. These options fit the idea of a low-cost model that can be used at home. This is a proof-of-concept study, and we did not measure learning curves, model validity, or detailed motion metrics. More precise analyses, such as surface area of perforations, fine-motion control or pressure measurement, would require equipment like microscope video recordings, sensors or specialized software, which lies outside the scope of a simple home-based training kit. Overall, the work remains preliminary and descriptive. 

## Conclusions

Bone drilling using a high-speed drill under magnification is an essential neurosurgical skill that needs to be practiced from early on by young trainees in order to refine this method and improve the safety and accuracy of this technique during various surgical procedures. Our simulation model, which includes the use of walnuts as a practice material and a battery-powered nail drill, provides a biologically safe, affordable, and reproducible method that could enhance drilling skills. It is notable that this material does not replicate the hardness of real bone and that the model does not use a surgical high-speed drill. Its role is therefore supplementary to already well-established methods and contributes primarily to familiarizing residents with the concept of the eggshell-peeling technique in the comfort of their personal environment, without the need for special equipment or expensive simulating models. We see this work as an early step, and future studies with more participants will be helpful to see how well the model performs in a broader training setting.
